# Positive association between triglyceride glucose index and arterial stiffness in hypertensive patients: the China H-type Hypertension Registry Study

**DOI:** 10.1186/s12933-020-01124-2

**Published:** 2020-09-18

**Authors:** Minghui Li, Aihua Zhan, Xiao Huang, Lihua Hu, Wei Zhou, Tao Wang, Lingjuan Zhu, Huihui Bao, Xiaoshu Cheng

**Affiliations:** 1 Department of Cardiovascular Medicine, The Second Affiliated Hospital of Nanchang University, No. 1 Minde Road, Nanchang, 330006 Jiangxi China; 2 Center for Prevention and Treatment of Cardiovascular Diseases, The Second Affiliated Hospital of Nanchang University, Nanchang, Jiangxi China; 3 Zheyuan township hospital, Wuyuan, Jiangxi China

**Keywords:** Triglyceride glucose index, Brachial-ankle pulse wave velocity, Arterial stiffness, Hypertension

## Abstract

**Background:**

Data are limited on whether TyG index is an independent predictor of arterial stiffness in hypertensive patients. The purpose of this study was to assess the association between the TyG index and arterial stiffness, and examined whether there were effect modifiers, in hypertensive patients.

**Methods:**

This study included 4718 hypertensive adults, a subset of the China H-type Hypertension Registry Study. The TyG index was calculated as ln[fasting triglycerides (mg/dL) × fasting glucose (mg/dL)/2]. Arterial stiffness was determined by measuring brachial-ankle pulse wave velocity (baPWV).

**Results:**

The overall mean TyG index was 8.84. Multivariate linear regression analyses showed that TyG index was independently and positively associated with baPWV (β, 1.02; 95% confidence interval [CI] 0.83, 1.20). Consistently, Multiple logistic analyses showed a positive association between TyG index risk of elevated baPWV (> 75th percentile) (odds ratio [OR], 2.12; 95% CI 1.80, 2.50). Analyses using restricted cubic spline confirmed that the associations of TyG index with baPWV and elevated baPWV were linear. Subgroup analyses showed that stronger associations between TyG index and baPWV were detected in men (all *P* for interaction < 0.05).

**Conclusion:**

TyG index was independently and positively associated with baPWV and elevated baPWV among hypertensive patients, especially in men. The data suggest that TyG index may serve as a simple and effective tool for arterial stiffness risk assessment in daily clinical practice.

## Background

Arterial stiffness, which is assessed by measuring pulse wave velocity (PWV), is recognized as an important predictor of cardiovascular events and mortality [1–3]. In clinical setting, Brachia-ankle pulse wave velocity (baPWV) measurement is widely used in large population studies, due to the advantages of high repeatability, simple operation and low time consumption, as well as it correlates with the stiffness of both central conduit and peripheral arteries. Most studies have revealed that elevated baPWV was associated with an increase in the risk of hypertension [4], diabetes [5], stroke [6], coronary artery calcification [7], cardiovascular disease (CVD) [3, 8], and mortality [9]. Hypertension is one of the major factors that leads to arterial stiffness. According to China Hypertension Survey (2012–2015) [10], 244.5 million Chinese adults were hypertension (prevalence 23.2%). Actually, the disturbances in glucose and lipid homeostasis are common in patients with hypertension. It has been reported that the prevalence of dyslipidemia and impaired fasting Glucose (IFG) were 41.9% and 40.7% in hypertensive patients [11, 12]. The coexistence of abnormal glycolipid metabolism in hypertensive patients greatly enhances the likelihood of these patients developing arterial stiffness. Therefore, a better understanding of the glycolipid metabolism factors among hypertensive patients may possibly reduce the huge burden of arterial stiffness associated complications.

Insulin resistance is a decrease in tissue response to insulin stimulation and is considered a key factor in the glycolipid metabolism [13]. Epidemiological and pathophysiological studies suggest that IR may be largely responsible for arterial stiffness progress and CVD [14–17]. The hyperinsulinemic euglycemic clamp is the ‘gold standard’ for evaluating insulin resistance [18]. However, this method of assessing IR is time consuming, expensive, complex, and requires an intensive labor force. Thus, it is not ideal for routine clinical applications. Recently, triglyceride-glucose (TyG) index was suggested as a reliable and inexpensive surrogate biomarker of IR. The TyG index was calculated as ln[fasting triglycerides (mg/dL) × fasting glucose (mg/dL)/2] and shows a direct correlation with hyperinsulinemic euglycemic clamp [19–21].

Several studies have demonstrated that higher levels of TyG index were associated with an increased risk of arterial stiffness [22–26]. However, most of the studies were conducted in a relatively healthy population. Little is known, about the role of TyG index, to assess the risk of arterial stiffness in hypertensive patients. Furthermore, few previous studies have comprehensively investigated potential modifiers of the association between TyG index and arterial stiffness risk. Therefore, we aimed to clarify whether TyG index could be used as an independent biomarker to predict the risk of arterial stiffness in hypertensive patients, and to examine any possible effect modifiers, using data from China H-type Hypertension Registry Study.

## Methods

### Participants

Data analyzed in this study was the baseline of the ongoing China H-type Hypertension Registry Study (Registration number: ChiCTR1800017274). The method of data collection and the exclusion criteria have been described previously [27]. Briefly, the study is a real-world, multicenter, observational study, conducted from Wuyuan, Jiangxi province of China, which conducted in March 2018. Eligible participants were adults aged 18 years and older who had hypertension, defined as seated, resting systolic blood pressure (SBP) ≥ 140 mmHg or diastolic blood pressure (DBP) ≥ 90 mmHg at the screening, or who were on antihypertensive medications. The exclusion criteria included neurological abnormalities, unable to be followed-up according to the study protocol, or plans to relocate shortly, and the patients, who are not suitable for inclusion or for long-term follow-up as assessed by study physicians. The study was conducted in accordance with the Declaration of Helsinki, and the protocol was approved by the Ethics Committee of Institute of Biomedicine, Anhui Medical University. All participants provided written informed consent.

This study was restricted to a subset of participants with baPWV data available at baseline (n = 5233). Participants with an ankle-brachial index (ABI) < 0.90 (n = 124), taking statins (n = 181), or using glucose-lowering medications (n = 210), were excluded. Finally, 4718 subjects were analyzed. The selection process of study analytic sample was detailed description in Additional file 1: Figure S1.

### Clinical characteristics

According to a standard operating procedure, the baseline data of clinical examination and detailed questionnaires were collected by trained researchers. The standard questionnaire included age, sex, education, physical activity, current medication from pill bottles, previous medical diagnoses, smoking history, and drinking history. Anthropometric parameters indicators of the clinical examination included weight, height, waist circumference, SBP, DBP. The body mass index (BMI) was calculated as weight (kg)/height (m^2^). Blood pressure (BP) was measured using an electronic sphygmomanometers (Omron; Dalian, China) with the subject in the sitting position after resting for 10 min, the average of the three measurements was used.

### Laboratory assays

Fasting venous blood samples were collected at the baseline, and were processed and analyzed at the clinical laboratory of the National Clinical Research Center for Kidney Disease, Guangzhou, China. Fasting plasma glucose, fasting lipids (total cholesterol, high-density lipoprotein-cholesterol (HDL-C), low-density lipoprotein cholesterol (LDL-C), and triglycerides), serum homocysteine, serum uric acid, and creatinine were determined using automatic clinical analyzers (Beckman Coulter). The TyG index was calculated as ln[fasting triglycerides (mg/dL) × fasting glucose (mg/dL)/2]. The formula for estimated glomerular filtration rate (eGFR) was used the Chronic Kidney Disease Epidemiology Collaboration (CKD-EPI) equation [28].

### BaPWV measurements

The ankle-brachial index (ABI) and baPWV were measured simultaneously with Omron Colin BP-203RPE III device (Omron Health Care, Kyoto, Japan). After having rest for more than 5 min in the supine position, 4 cuffs were wrapped around bilateral brachia and ankles and connected to a plethysmographic sensor and oscillometric pressure sensor. ABI was measured by the ankle SBP divided by the brachial SBP. Pressure waveforms were recorded using semiconductor pressure sensors to assess the transmission time between the initial rises in both the brachial and tibial arteries waves. The distance between sampling points of baPWV was estimated based on height. The baPWV was calculated using the formula (La-Lb)/Tba. La is the distance from the heart to the ankle, Lb is the distance from the heart to the brachium, and Tba is the time interval between the brachial and ankle waveforms. Two trained technicians were performed twice, and the average values of the left and right side assessments was used as a marker of arterial stiffness.

### Statistical analysis

Data are presented as mean ± standard deviation (SD) for continuous variables and as frequency (%) for categorical variables. The baseline characteristics of the different groups by TyG index quartiles were compared using ANOVA tests or Chi square tests. The relationships between the TyG index and cardiometabolic risk factors were examined using Pearson’s correlation. Because the predictive value of baPWV in cardiovascular events was not available. Elevated baPWV was defined as a value greater than 75th percentile of baPWV value in present study, which was greater than 20.02 m/s. The independent association of the TyG index with baPWV and elevated baPWV were evaluated using multivariate linear regression models (beta coefficient [β] and 95% confidence interval [CI]) and multivariate logistic regression models (odds ratio [OR] and 95% CI) with adjustment for major covariables in three models. Model 1: adjusted for age; Model 2: further adjusted for age, sex, education, BMI, waist circumference, physical activity, current smoking, current drinking, SBP, DBP; Model 3: additionally adjusted for age, sex, education, BMI, waist circumference, physical activity, current smoking, current drinking, SBP, DBP, serum uric acid, serum homocysteine, HDL-C, LDL-C, eGFR, self-reported diabetes, antihypertensive drugs, antiplatelet drugs. In the regression analyses model, the following variables were selected because of their clinical importance, statistical significance in the univariable analysis, and the potential confounders effect estimates individually changed by at least 10%. Dose–response association of TyG index with baPWV and elevated baPWV were conducted using generalized additive model (GAM) and a fitted smoothing curve (penalized spline method). In addition, possible modifications on the association between TyG index and baPWV were also evaluated by stratified analyses and interaction testing.

All data analyzed were using the statistical package R (http://www.r-project.org) and Empower (R) (www.empowerstats.com; X&Y Solutions, Inc., Boston, MA). A 2-tailed P < 0.05 was considered to be statistically significant.

## Results

### Baseline characteristics

Data of 4718 participants were included in current analysis. The mean (SD) age of the participants was 64.41 (9.48) years; 2346 were men (49.72%). The mean baseline TyG index was 8.84 (0.63). Mean baPWV was 18.05 (3.85) m/s. The characteristics of the participants by TyG index quartiles are presented in Table 1. TyG index was inversely associated with age, serum homocysteine, HDL-C, and positively associated with BMI, waist circumference, DBP, fasting plasma glucose, total cholesterol, triglycerides, serum uric acid, LDL-C, and eGFR. Moreover, participants with higher TyG index were significantly more likely to have diabetes mellitus, higher education levels, and using antihypertensive drugs. Participants with lower TyG index were significantly more likely to men, current smokers, and current drinkers.

**Table 1:** Clinical characteristics of the study population according to TyG index

	Q1	Q2	Q3	Q4	P value
N	1180	1179	1179	1180	
Age (years)	66.93 ± 9.38	65.30 ± 9.48	63.86 ± 9.16	61.56 ± 9.07	< 0.001
Male, n (%)	765 (64.83)	588 (49.87)	493 (41.82)	500 (42.37)	< 0.001
Education, n (%)					< 0.001
< High school	1080 (91.53)	1079 (91.52)	1061 (89.99)	1030 (87.29)	
≥ High school	100 (8.47)	100 (8.48)	118 (10.01)	150 (12.71)	
BMI (kg/m^2^)	21.43 ± 3.04	22.71 ± 3.35	23.85 ± 3.31	24.95 ± 3.23	< 0.001
Waist circumference (cm)	77.05 ± 8.85	80.74 ± 9.29	83.69 ± 8.85	86.78 ± 8.43	< 0.001
Physical activity, n (%)					0.283
Mild	610 (51.69)	625 (53.01)	642 (54.45)	651 (55.17)	
Moderate	293 (24.83)	258 (21.88)	273 (23.16)	275 (23.31)	
Vigorous	277 (23.47)	296 (25.11)	264 (22.39)	254 (21.53)	
SBP (mmHg)	147.10 ± 20.54	147.45 ± 19.01	147.26 ± 16.91	147.93 ± 17.39	0.717
DBP (mmHg)	87.79 ± 14.53	88.85 ± 13.80	89.00 ± 10.59	91.05 ± 10.82	<0.001
Fasting plasma glucose (mmol/L)	5.36 ± 0.60	5.71 ± 0.71	6.05 ± 0.99	6.93 ± 2.09	< 0.001
Total cholesterol (mmol/L)	4.58 ± 0.89	5.04 ± 0.93	5.39 ± 1.04	5.57 ± 1.23	< 0.001
Triglyceride (mmol/L)	0.80 ± 0.16	1.21 ± 0.18	1.71 ± 0.30	3.24 ± 1.65	< 0.001
Serum uric acid, ummol/L	407.56 ± 111.40	415.11 ± 117.35	432.64 ± 119.33	468.18 ± 125.63	< 0.001
Serum homocysteine, μmol/L	18.79 ± 11.99	19.34 ± 12.78	18.24 ± 11.70	17.72 ± 10.27	0.006
HDL-C (mmol/L)	1.63 ± 0.43	1.55 ± 0.39	1.47 ± 0.35	1.34 ± 0.36	< 0.001
LDL-C (mmol/L)	2.43 ± 0.58	2.82 ± 0.64	3.16 ± 0.75	3.34 ± 0.84	< 0.001
eGFR (mL/min per 1.73 m^2^)	85.63 ± 19.62	86.00 ± 19.25	86.88 ± 18.28	87.77 ± 19.39	0.017
TyG index	8.11 ± 0.23	8.59 ± 0.11	8.99 ± 0.12	9.68 ± 0.41	< 0.001
Current smoking, n (%)	455 (38.56)	322 (27.31)	276 (23.41)	294 (24.92)	< 0.001
Current drinking, n (%)	359 (30.42)	291 (24.68)	249 (21.12)	282 (23.90)	< 0.001
Self-reported diabetes, n (%)	26 (2.20)	37 (3.14)	47 (3.99)	122 (10.34)	< 0.001
Antihypertensive drugs, n (%)	657 (55.68)	710 (60.22)	721 (61.15)	731 (61.95)	0.009
Antiplatelet drugs, n (%)	18 (1.53)	29 (2.46)	29 (2.46)	20 (1.69)	0.226

Data are the mean ± SD, or number (percentage)

*BMI* body mass index, *SBP* systolic blood pressure, *DBP* diastolic blood pressure, *HDL*-*C* high-density lipoprotein cholesterol, *LDL*-*C* low-density lipoprotein cholesterol, *eGFR* estimated glomerular fltration rate, *TyG* triglyceride glucose

### Correlation between the TyG index and cardiometabolic risk factors

The results of pearson’s correlation analysis for the relationship between the TyG index and cardiometabolic risk factors were described in Table 2. The TyG index was significantly correlated with BMI, waist circumference, SBP, DBP, HDL-C, LDL-C, serum uric acid, and eGFR, but not serum homocysteine, after adjusted for age and sex.

**Table 2:** The correlation between TyG index and cardiometabolic risk factors after adjusted for age and sex

	R	P value
BMI (kg/m^2^)	0.335	< 0.001
Waist circumference (cm)	0.373	< 0.001
SBP (mmHg)	0.030	0.042
DBP (mmHg)	0.055	< 0.001
Total cholesterol (mmol/L)	0.316	< 0.001
HDL-C (mmol/L)	− 0.284	< 0.001
LDL-C (mmol/L)	0.395	< 0.001
Serum uric acid (umol/L)	0.291	< 0.001
eGFR (mL/min per 1.73 m^2^)	− 0.095	< 0.001
Serum homocysteine (μmol/L)	0.021	0.158

*TyG* triglyceride glucose, *BMI* body mass index, *SBP* systolic blood pressure, *DBP* diastolic blood pressure, *HDL*-*C* high-density lipoprotein cholesterol, *LDL*-*C* low-density lipoprotein cholesterol *eGFR* estimated glomerular fltration rate

### Association of TyG index with baPWV and elevated baPWV

**Fig. 1:**
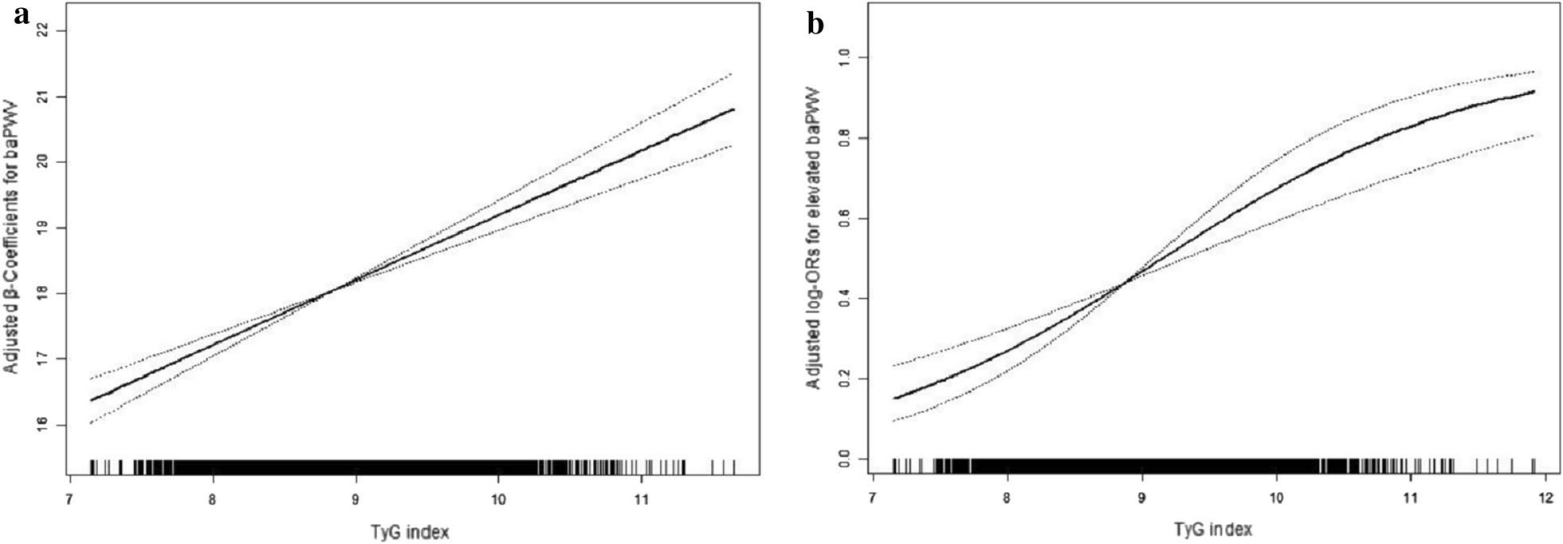
Dose–response relationship between TyG index and baPWV*. **a** TyG index and baPWV;** b** TyG index and elevated baPWV. *Adjusted for age, sex, education, BMI, waist circumference, physical activity, current smoking, current drinking, SBP, DBP, serum uric acid, serum homocysteine, HDL-C, LDL-C, eGFR, self-reported diabetes, antihypertensive drugs, antiplatelet drugs. Abbreviations: *TyG* triglyceride glucose, *ba*-*PWV* brachial to ankle pulse wave velocity

Overall, there were significant positive associations of TyG index with baPWV and the risk of elevated baPWV (Fig. 1a, b). Per 1 unit increment in TyG index, baPWV is changed in 1.02 m/s (95% CI 0.83, 1.20) according to the estimation from regression coefficients indication, and the odds ratios (OR) of the risk of elevated baPWV was 2.12 (95% CI 1.80, 2.50).

As shown in Table 3, after adjustment for different confounders, the positive association between TyG index and baPWV was found in all models (model 1–3). When TyG index was assessed as quartiles, in the fully adjusted model (model 3), the adjusted β of baPWV for participants in quartile 2, quartile 3, and quartile 4 were 0.59 (95% CI 0.33, 0.85), 0.89 (95% CI 0.61, 1.18), and 1.56 (95% CI 1.25, 1.88), respectively, compared with those in quartile 1 (*P* for trend < 0.001). Consistently, Table 4 presented the relative odds of having an elevated baPWV. Compared to participants in the lowest TyG index quartile, the adjusted OR for participants in the second, third and fourth quartiles were 1.49 (95% CI 1.18, 1.88), 2.07 (95% CI 1.60, 2.67), and 3.35 (95% CI 2.52, 4.45), respectively. Also, *P* for trend in all models was significant (*P* for trend < 0.001), suggesting a dose–response relation between TyG index and baPWV and elevated baPWV.

**Table 3:** Association between TyG index and baPWV in different models

TyG index	BaPWV, m/s, β (95%CI)		
	Model 1	Model 2	Model 3
Per 1 unit increase	0.86 (0.70, 1.02)	0.94 (0.78, 1.10)	1.02 (0.83, 1.20)
Quartiles			
Q1 (≥ 7.15, < 8.40)	0.00	0.00	0.00
Q2 (≥ 8.40, < 8.78)	0.56 (0.28, 0.84)	0.57 (0.31, 0.82)	0.59 (0.33, 0.85)
Q3 (≥ 8.78, < 9.22)	0.72 (0.44, 1.00)	0.83 (0.56, 1.09)	0.89 (0.61, 1.18)
Q4 (≥ 9.22, ≤ 11.65)	1.37 (1.09, 1.65)	1.48 (1.21, 1.76)	1.56 (1.25, 1.88)
*P* for trend	0.021	<0.001	<0.001

Model 1: adjusted age

Model 2: adjusted for age, sex, education, BMI, waist circumference, physical activity, current smoking, current drinking, SBP, DBP

Model 3: adjusted for age, sex, education, BMI, waist circumference, physical activity, current smoking, current drinking, SBP, DBP, serum uric acid, serum homocysteine, HDL-C, LDL-C, eGFR, self-reported diabetes, antihypertensive drugs, antiplatelet drugs

*TyG* triglyceride glucose, *ba*-*PWV* brachial to ankle pulse wave velocity, CI confidence interval

**Table 4:** Association between TyG index and elevated baPWV in different models

TyG index	Elevated baPWV, OR (95%CI)		
	Model 1	Model 2	Model 3
Per 1 unit increase	1.77 (1.57, 1.99)	2.03 (1.76, 2.34)	2.12 (1.80, 2.50)
Quartiles			
Q1 (≥ 7.15, < 8.40)	1.00	1.00	1.00
Q2 (≥ 8.40, < 8.78)	1.33 (1.08, 1.63)	1.46 (1.16, 1.83)	1.49 (1.18, 1.88)
Q3 (≥ 8.78, < 9.22)	1.63 (1.32, 2.00)	1.96 (1.55, 2.49)	2.07 (1.60, 2.67)
Q4 (≥ 9.22, ≤ 11.65)	2.47 (2.00, 3.05)	3.16 (2.46, 4.05)	3.35 (2.52, 4.45)
*P* for trend	< 0.001	< 0.001	< 0.001

Model 1: adjusted age

Model 2: adjusted for age, sex, education, BMI, waist circumference, physical activity, current smoking, current drinking, SBP, DBP

Model 3: adjusted for age, sex, education, BMI, waist circumference, physical activity, current smoking, current drinking, SBP, DBP, serum uric acid, serum homocysteine, HDL-C, LDL-C, eGFR, self-reported diabetes, antihypertensive drugs, antiplatelet drugs

*TyG* triglyceride glucose, *ba*-*PWV* brachial to ankle pulse wave velocity, CI confidence interval

### Subgroup analyses by potential effect modifiers

We further performed stratified analyses to assess the effect of TyG index (per 1 unit increment) on baPWV in various subgroups. The association between TyG index and baPWV were consistent in the following subgroups: age (< 65 vs. ≥ 65 y; *P*-interaction = 0.11), BMI (< 25 vs. ≥ 25 kg/m^2^; *P*-interaction = 0.15), physical activity (mild, moderate, vigorous; *P*-interaction = 0.71), current smoking (no vs. yes; *P*-interaction = 0.37), current alcohol drinking (no vs. yes; *P*-interaction = 0.97), SBP (< 140, 140-159, ≥ 160 mmHg; *P*-interaction = 0.99), DBP (< 90, 90-99, ≥ 100 mmHg; *P*-interaction = 0.30), self-reported diabetes (no vs. yes; *P*-interaction = 0.68), LDL-C (median, < 2.85 vs. ≥ 2.85 mmol/L; *P*-interaction = 0.38), and eGFR (< 60 vs. ≥ 60 mL/min per 1.73 m^2^; *P*-interaction = 0.55).

**Fig. 2:**
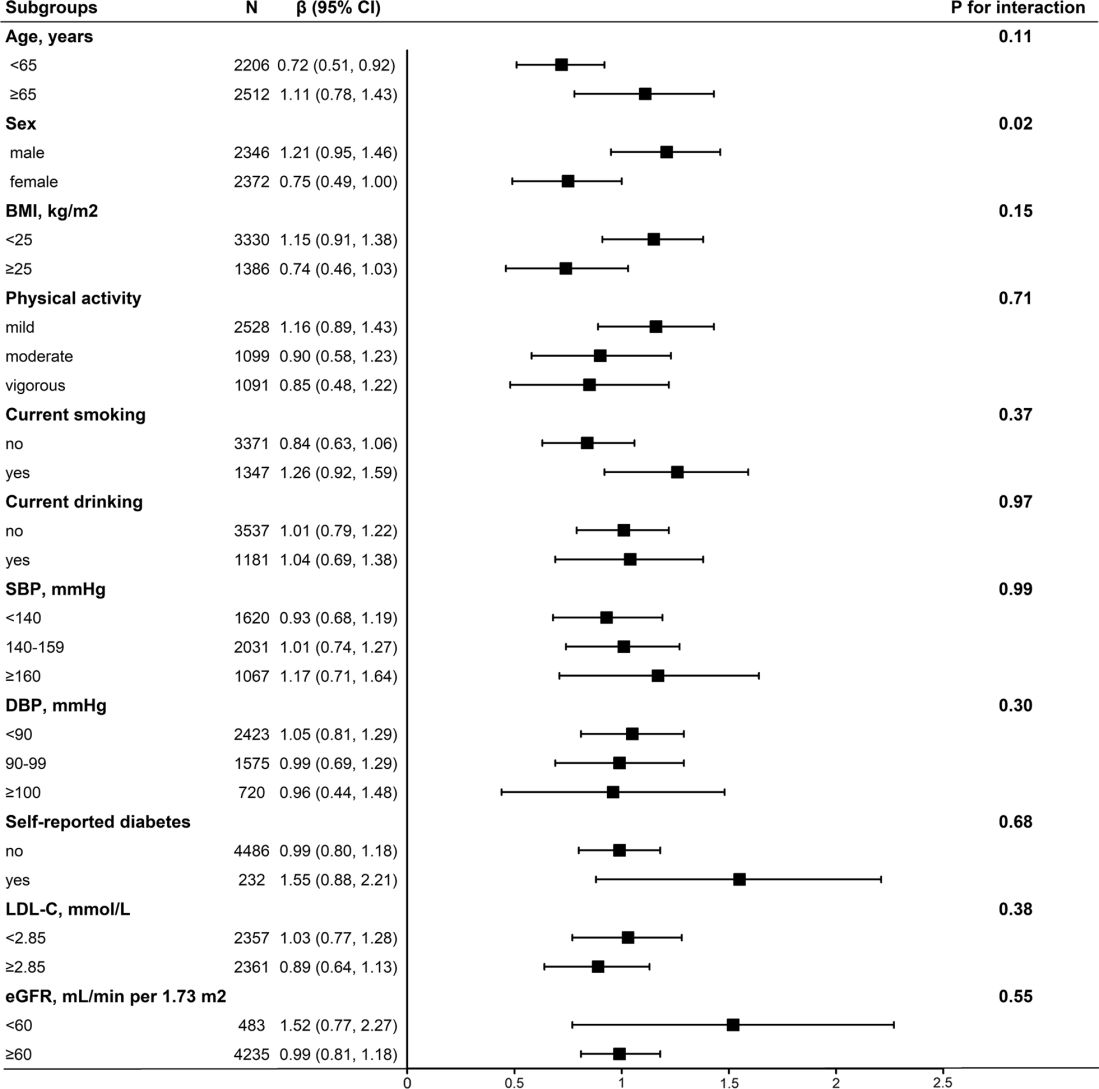
Subgroup analyses of the effect of TyG index on baPWV. Each subgroup analysis adjusted, if not stratified, for age, sex, education, BMI, waist circumference, physical activity, current smoking, current drinking, SBP, DBP, serum uric acid, serum homocysteine, HDL-C, LDL-C, eGFR, self-reported diabetes, antihypertensive drugs, antiplatelet drugs. Abbreviations: *TyG* triglyceride glucose, *ba*-*PWV* brachial to ankle pulse wave velocity, *BMI* body mass index, *LDL*-*C* low-density lipoprotein cholesterol, *eGFR* estimated glomerular fltration rate, *CI* confidence interval

However, there was a significant interaction between TyG index and sex on baPWV. A stronger positive association between TyG index and baPWV was found in men (β, 1.21; 95% CI 0.95, 1.46) compared with women (β, 0.75; 95% CI 0.49, 1.00; *P*-interaction = 0.02) (Fig. 2, Additional file 1: Figure S2). In addition, We further converted TyG index from a continuous variable to a categorical variable (quartiles) to explored the association of TyG index with baPWV and elevated baPWV in different gender. The Magnitude of the effect of increase of the TyG index on baPWV and elevated baPWV were greater in men than in women (Additional file 1: Table S1).

## Discussion

This study lends further support to the growing literature pointing to TyG index as an independent predictor of arterial stiffness, we demonstrated a significant positive association between TyG index and baPWV in hypertensive patients. Furthermore, our study expands the results of previously published studies by demonstrating that the positive relation of TyG index with baPWV was more pronounced in men.

Several studies have reported a positive association of TyG index with arterial stiffness and CVD [23–26, 29–31]. Irace et al. [26] used data from two different cohorts found that the TyG index was strongly associated with carotid atherosclerosis, which was assessed by Doppler ultrasonography, and had better predictive value for IR than the homeostatic model assessment of insulin resistance (HOMA-IR). Won et al. [25] conducted a cross-sectional study of 2560 Korean subjects demonstrated that the TyG index is independently associated with arterial stiffness in a relatively healthy population. A recent study in a Northern Shanghai Study of China also showed that an elevated TyG index was significantly associated with a higher risk of arterial stiffness and nephric microvascular damage after adjustment for traditional cardiovascular risk factors [24]. As well all known, hypertension is a major risk factor for arterial stiffness. Cross-sectional and cohort studies have also founded that TyG index is an independent risk factor for the risk of hypertension [32, 33]. However, for high risk arterial stiffness hypertensive population, there is a particular lack of data on the role of TyG index on arterial stiffness. While in our study, we reported for the first time to examine the relationship between TyG index and arterial stiffness in hypertensive patients. Our analysis also clearly demonstrated that the TyG index was independently and positively associated with baPWV, even when the subjects included in the analysis were limited to those with high risk of arterial stiffness. Further confirmed the possibility that TyG index may serve as a novel and simple noninvasive biomarker to help evaluate the risk of arterial stiffness.

This study has also provide an another new insights, as a key finding to the sex-TyG index interaction. To our knowledge, only two studies have examined the association between TyG index and sex on arterial stiffness. Lee et al. [23] conducted a cross-sectional study of 3587 relatively healthy Korean adults, the results showed that the TyG index was independently associated with increased arterial stiffness, the OR for increased baPWV (> 75th percentile) for the highest and lowest quartiles of the TyG index was 2.92 (95% CI 1.92–4.44) in men and 1.84 (95% CI 1.15–2.96) in women. Consistent with the above results, our study also found that the magnitude of the effect of increase of the TyG index on the risk of elevated baPWV was greater in men than women (Additional file 1: Table S1). However, another study by Nakagomi et al. [34] examined the association between surrogate markers of insulin resistance and arterial stiffness in 2818 participants, this study found that the TyG index was associated with increased baPWV (> 75th percentile) and the associations were stronger in women than in men. The conflicting results might be attributed to the differences in age distribution. The mean age of the participants in Nakagomi et al. study was 38.8 years, while it was approximately 64.41 years in our study.

Although the exact mechanisms underlying the relationship of the TyG index with arterial stiffness is unclear, it may be linked to IR. IR is associated with hyperinsulinaemia, hyperglycaemia, dyslipidemia, hypertension and a proinflammatory state, as well as the effect of perturbed insulin signaling at the level of the intimal cells (endothelial cells, vascular smooth muscle cells, and macrophages), all of which predispose to arterial stiffness [13, 14, 35–37]. In our study, men showed larger regression coefficients and odds ratios of the TyG index in relation with increased arterial stiffness than women. The possible explanation for this inconsistency is the sex difference in some specific contributing factors to IR. Compared with women, men have more risk factors related to metabolic diseases. Such as, men were more likely to be smokers and drinkers, and had higher WC, serum uric acid, serum homocysteine, and lower eGFR (Additional file 1: Table S2). Further research is needed to examine the relationship between sex, IR and arterial stiffness.

The strengths of the present study were the inclusion of a large number of hypertensive participants, adjustment to minimize residual confounders, handled target independent variables as both continuous variables and categorical variables to reduce the contingency in the data analysis, and the subgroup analyses. However, several limitations should also be noted. First, this study was a cross-sectional design, which limits detection of causality. Second, the HOMA-IR of IR was not calculated because insulin levels were rarely detected in large epidemiological investigations. However, the direct correlation between the TyG index and HOMA-IR was well-established. Third, we did not collected detailed food intake information and inflammation indicators at baseline. As such, we could not examine the possible modifying effect of different foods on the TyG index-arterial stiffness association. Fourth, Impaired glucose homeostasis and inflammatory may play an important role in arterial stiffness and IR. In our study, we did not detect 2-h oral glucose tolerance test and inflammatory indicators. Fifth, all the participants included in this study were Chinese hypertensive patients. Therefore, it would be difficult to generalize to other populations.

## Conclusion

In subjects with hypertension, we found a significant positive association between TyG index and baPWV, especially in men. The data suggest that TyG index may serve as a simple and effective tool for arterial stiffness risk assessment in daily clinical practice.

## Supplementary information

**Additional file 1: Fig. S1.** Flow chart of the study participants**. Fig. S2.** Dose–response relationship between TyG index and baPWV in different gender. *Adjusted for age, education, BMI, waist circumference, physical activity, current smoking, current drinking, SBP, DBP, serum uric acid, serum homocysteine, HDL-C, LDL-C, eGFR, self-reported diabetes, antihypertensive drugs, antiplatelet drugs. Abbreviations: *TyG* triglyceride glucose, *ba*-*PWV* brachial to ankle pulse wave velocity.

## Data Availability

The datasets used and/or analyzed in the current study are available from the corresponding author upon reasonable request.

## Abbreviations

PWV: Pulse wave velocity
ba-PWV: Brachial-ankle pulse wave velocity
CVD: Cardiovascular disease
IR: Insulin resistance
TyG: Triglyceride glucose
SBP: Systolic blood pressure
DBP: Diastolic blood pressure
BMI: Body mass index
TC: Total cholesterol
HDL-C: High-density lipoprotein cholesterol
TG: Triglycerides
SUA: Serum uric acid
LDL-C: Low-density lipoprotein cholesterol
eGFR: Estimated glomerular filtration rate
ABI: Ankle–brachial index
BP: Blood pressure
SD: Standard deviation
CI: Confidence interval
OR: Odds ratio
β: Beta coefficient
HOMA-IR: Homeostasis model assessment of insulin resistance
