# Supplementary material for: Positive association between triglyceride glucose index and arterial stiffness in hypertensive patients: the China H-type Hypertension Registry Study
Source: Cardiovasc Diabetol. 2020 Sep 18;19:139. doi: 10.1186/s12933-020-01124-2 (PMC7501677; doi:10.1186/s12933-020-01124-2)
Supplement: Supplementary file 1 — Additional file 1: Fig. S1. Flow chart of the study participants. Fig. S2. Dose–response relationship between TyG index and baPWV in different gender. *Adjusted for age, education, BMI, waist circumference, physical activity, current smoking, current drinking, SBP, DBP, serum uric acid, serum homocysteine, HDL-C, LDL-C, eGFR, self-reported diabetes, antihypertensive drugs, antiplatelet drugs. Abbreviations: TyG triglyceride glucose, ba-PWV brachial to ankle pulse wave velocity. [file 12933_2020_1124_MOESM1_ESM.docx]

5233 participants with brachial-ankle pulse wave velocity (baPWV) available data at baseline

Excluded n = 515

ABI < 0.90, n = 124;

Taking statins, n = 181;

Using glucose-lowering medications, n = 210.

4718 subjects were analyzed

TyG index Q4

(≥9.22, ≤11.65), n = 1180

TyG index Q3

(≥8.78, <9.22), n = 1179

TyG index Q2

(≥8.40, <8.78), n = 1179

TyG index Q1

(≥7.15, <8.40) , n = 1180

**Fig. S1 Flow chart of the study participants**


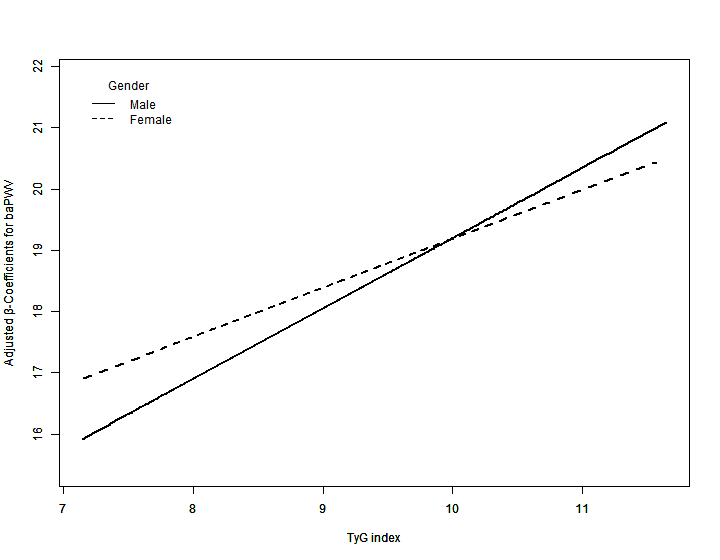


**Fig. S2 Dose–response relationship between TyG index and baPWV in different gender**

*Adjusted for age, sex, education, BMI, waist circumference, physical activity, current smoking, current drinking, SBP, DBP, serum uric acid, serum homocysteine, HDL-C, LDL-C, eGFR, diabetes mellitus, antihypertensive drugs, antiplatelet drugs.


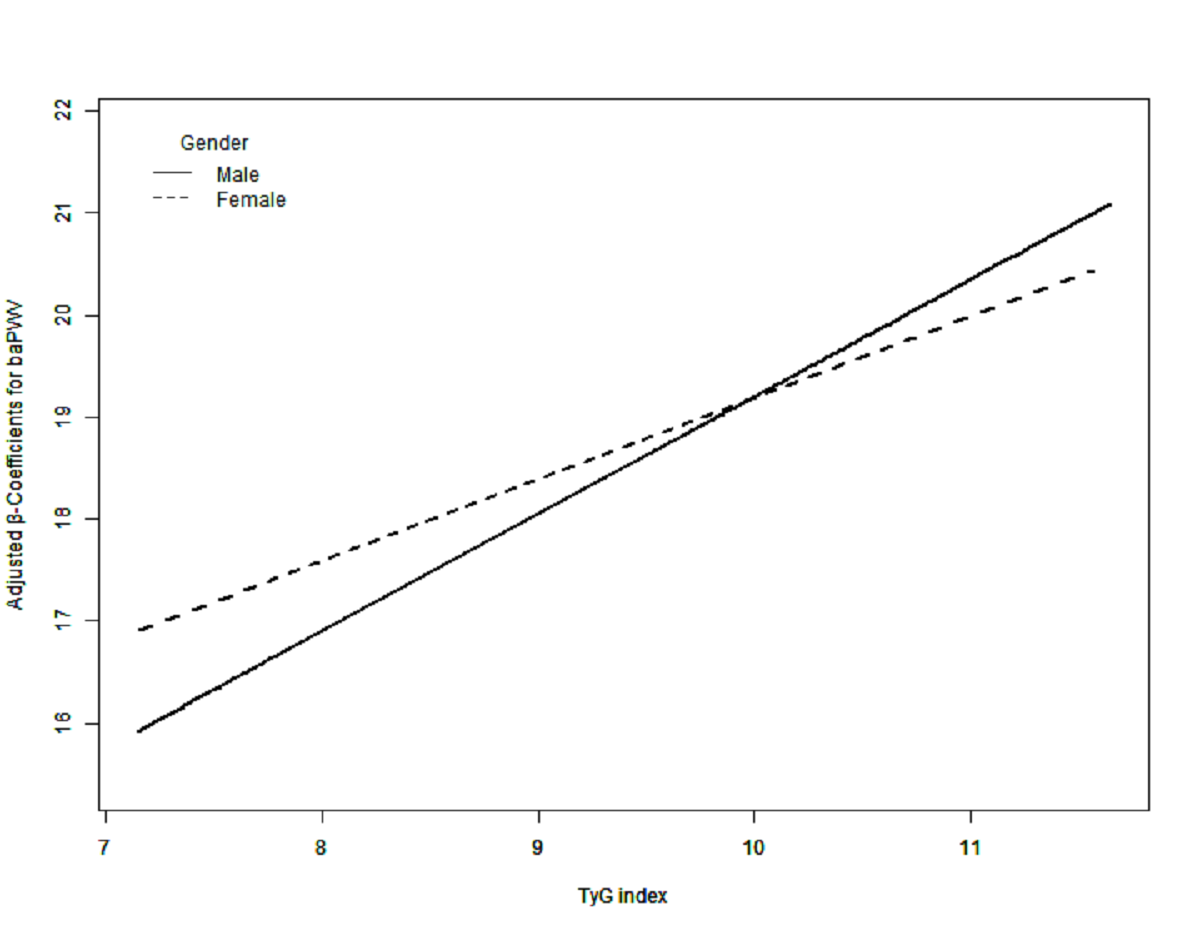


**Table S1. The relationship between TyG index and baPWV in different gender**

| TyG index | baPWV, m/s, β (95%CI) | |
| --- | --- | --- |
|  | Male | Female |
| Per 1 unit increase | 1.21 (0.95, 1.46) | 0.75 (0.49, 1.00) |
| Quartiles |  |  |
| Q1 (≥7.15, <8.40) | 0 | 0 |
| Q2 (≥8.40, <8.78) | 0.68 (0.33, 1.02) | 0.40 (0.00, 0.79) |
| Q3 (≥8.78, <9.22) | 1.08 (0.69, 1.48) | 0.58 (0.17, 0.99) |
| Q4 (≥9.22, ≤11.65) | 1.93 (1.49, 2.38) | 1.06 (0.61, 1.51) |
| P for trend | <0.001 | <0.001 |
|  | Elevated baPWV OR (95%CI)* | |
|  | Male | Female |
| Per 1 unit increase | 2.35 (1.86, 2.97) | 1.84 (1.46, 2.32) |
| Quartiles |  |  |
| Q1 (≥7.15, <8.40) | 1 | 1 |
| Q2 (≥8.40, <8.78) | 1.56 (1.15, 2.12) | 1.49 (1.03, 2.16) |
| Q3 (≥8.78, <9.22) | 2.20 (1.55, 3.13) | 1.97 (1.34, 2.89) |
| Q4 (≥9.22, ≤11.65) | 3.87 (2.59, 5.80) | 2.71 (1.79, 4.12) |
| P for trend | <0.001 | <0.001 |

*Elevated baPWV was defned as 19.68 m/s for men and 20.34 m/s for women. Adjusted for age, sex, education, BMI, waist circumference, physical activity, current smoking, current drinking, SBP, DBP, serum uric acid, serum homocysteine, HDL-C, LDL-C, eGFR, diabetes mellitus, antihypertensive drugs, antiplatelet drugs.

Abbreviations: *TyG* triglyceride glucose, *ba-PWV* brachial to ankle pulse wave velocity, CI confidence interval

**Table S2 Baseline cardiometabolic risk factors of the study participants by gender**

|  | **Male (2346)** | **Female (2372)** | **P-value** |
| --- | --- | --- | --- |
| Age (years) | 64.42 ± 9.81 | 64.41 ± 9.15 | 0.987 |
| BMI (kg/m^2^) | 23.04 ± 3.40 | 23.43 ± 3.57 | <0.001 |
| Waist circumference (cm) | 82.77 ± 9.64 | 81.36 ± 9.43 | <0.001 |
| SBP (mmHg) | 145.79 ± 19.80 | 149.06 ± 17.00 | <0.001 |
| DBP (mmHg) | 90.50 ± 14.30 | 87.86 ± 10.52 | <0.001 |
| Total cholesterol (mmol/L) | 4.93 ± 1.06 | 5.35 ± 1.09 | <0.001 |
| Serum uric acid, ummol/L | 476.48 ± 118.50 | 385.77 ± 105.08 | <0.001 |
| Serum homocysteine, μmol/L | 20.99 ± 14.12 | 16.08 ± 8.05 | <0.001 |
| HDL-C (mmol/L) | 1.47 ± 0.40 | 1.53 ± 0.39 | <0.001 |
| LDL-C (mmol/L) | 2.81 ± 0.77 | 3.07 ± 0.79 | <0.001 |
| eGFR (mL/min per 1.73 m^2^) | 84.42 ± 19.89 | 88.69 ± 18.15 | <0.001 |
| Current smoking, n (%) | 1173 (50.00) | 174 (7.34) | <0.001 |
| Current drinking, n (%) | 1003 (42.75) | 178 (7.50) | <0.001 |

Data are the mean± SD, or number (percentage).

*BMI* body mass index, *SBP* systolic blood pressure, *DBP* diastolic blood pressure, *HDL-C* high-density lipoprotein cholesterol, *LDL-C* low-density lipoprotein cholesterol.
